# Deletion of TRAAK Potassium Channel Affects Brain Metabolism and Protects against Ischemia

**DOI:** 10.1371/journal.pone.0053266

**Published:** 2012-12-28

**Authors:** Christophe Laigle, Sylviane Confort-Gouny, Yann Le Fur, Patrick J. Cozzone, Angèle Viola

**Affiliations:** Centre de Résonance Magnétique Biologique et Médicale (CRMBM), Joint Research Unit n°7339 (UMR 7339), National Center for Scientific Research (CNRS), Aix-Marseille Université (AMU), Marseille, France; Albany Medical College, United States of America

## Abstract

Cerebral stroke is a worldwide leading cause of disability. The two-pore domain K^+^ channels identified as background channels are involved in many functions in brain under physiological and pathological conditions. We addressed the hypothesis that TRAAK, a mechano-gated and lipid-sensitive two-pore domain K^+^ channel, is involved in the pathophysiology of brain ischemia. We studied the effects of TRAAK deletion on brain morphology and metabolism under physiological conditions, and during temporary focal cerebral ischemia in Traak^−/−^ mice using a combination of *in vivo* magnetic resonance imaging (MRI) techniques and multinuclear magnetic resonance spectroscopy (MRS) methods. We provide the first *in vivo* evidence establishing a link between TRAAK and neurometabolism. Under physiological conditions, Traak^−/−^ mice showed a particular metabolic phenotype characterized by higher levels of taurine and *myo*-inositol than Traak^+/+^ mice. Upon ischemia, Traak^−/−^ mice had a smaller infarcted volume, with lower contribution of cellular edema than Traak^+/+^ mice. Moreover, brain microcirculation was less damaged, and brain metabolism and pH were preserved. Our results show that expression of TRAAK strongly influences tissue levels of organic osmolytes. Traak^−/−^ mice resilience to cellular edema under ischemia appears related to their physiologically high levels of *myo*-inositol and of taurine, an aminoacid involved in the modulation of mitochondrial activity and cell death. The beneficial effects of TRAAK deletion designate this channel as a promising pharmacological target for the treatment against stroke.

## Introduction

Potassium (K^+^) channels are involved in many cellular functions and considered as promising pharmacological targets for the treatment of neurodegenerative diseases and cerebral stroke, a worldwide leading cause of disability [1]. The two-pore domain K^+^ channels (K_2_P) allow passive K^+^ transmembranous transport and are involved in cell volume regulation [2]. In the central nervous system, they participate to neuronal K^+^ release and spatial glial K^+^ buffering [3]. They are designated as the background K^+^ channels maintaining resting membrane potential and are quasi-insensitive to classic K^+^ blockers [2], [4], [5]. They are viewed as important targets for modulation of neuronal activity [6]. Despite common structural features, K_2_P channels, which are widely distributed in the central nervous system [7], [8], [9] and cerebral arteries [10], show low sequence homology and diverse regulatory mechanisms [6], including temperature, pH, oxygen tension, osmolarity and/or membrane stretch [6], [11]. Among K_2_P channels, TREK channels, which are regulated by neurotransmitters and hormones [2], [5], form the first identified lipid- and stretch-activated K_2_P channels, with three members TREK-1 (K2P2.1 or KCNK2), TREK-2 (K2P10.1 or KCNK10) and TRAAK (K2P4.1 or KCNK4) [6]. Because these channels are activated by potent neuroprotectors such as polyunsaturated fatty acids (PUFA), it has been suggested that they could all be involved in neuroprotection [6]. Indeed, activation of TREK-1 channels, which are located at the pre and post-synaptic levels, by PUFA leads to a decrease in glutamatergic transmission in models of brain and spinal chord ischemia, and of epilepsy [12], [13]. However, the mechanisms by which PUFA activate TRAAK would be different from those involved in other TREK channels [14]. Recently, the implication of TREK-1 has been identified in several neurological conditions including ischemia [6], [12].

TRAAK, the first cloned PUFA- and stretch-activated K_2_P with unique functional properties [4], has been found in the human and the rodent brains [8], [9], with an important degree of conservation between humans and rodents in the cortex [8]. In the mouse brain, TRAAK mRNA is expressed in the entire hippocampus [15]. At the cellular level, TRAAK has been localized to neurons [15], but not to astrocytes [7]. TRAAK displays a specific electrophysiological behavior. Its current-voltage (I–V) relationship shows an outward rectification that can be approximated by the Goldman-Hodgkin-Katz current equation predicting specific curvature of the I–V plot in asymmetric K^+^ conditions, when external [K^+^] is low [16], [17]. This result suggests that TRAAK does not show any voltage sensitivity, a specific feature of background conductances. TRAAK is opened by membrane stretch, cell swelling, arachidonic acid [7], [18] and closed by hyperosmolarity [5]. Unlike TREK-1 and TREK-2 channels, TRAAK is strongly sensitive to internal alkalosis but not to acidosis, and a synergistic effect between mechanosensitivity and alkalosis has been shown [14]. Moreover, whilst Gα_q_ mediated inhibition of TREK-1 and TREK-2 has been demonstrated [19], there is only slight inhibition of TRAAK by this pathway [19], [20]. The physiological role of TRAAK within the brain is undefined, and the impact of TRAAK deletion on the cerebral phenotype, although essential to understanding its function, is unknown.

*In vivo* magnetic resonance imaging (MRI) and spectroscopy (MRS) are non-invasive techniques that permit longitudinal studies of structural, functional and metabolic alterations associated with brain diseases. They provide the opportunity of comparative investigations in humans and animals as they measure the same endpoints to evaluate disease progression. MRI is considered as the gold standard for identification of ischemic tissue in stroke, and for differentiation of irreversibly infarcted core from hypoperfused but salvageable penumbra [21]. Furthermore, the association of MRI to MRS, a technique enabling the study of cellular metabolism, appears more predictive of stroke outcome than MRI alone [22], [23].

We have undertaken a study dissecting the effects of TRAAK deletion on brain using *in vivo* brain MRI and multinuclear MRS, which may help elucidating connections between genes and metabolic phenotypes in transgenic mice [24]. We addressed the hypothesis that TRAAK is involved in the pathophysiology of brain ischemia by studying a model of transient occlusion of the middle cerebral artery (tMCAO) [6], [12]. Our results demonstrate that TRAAK strongly influences tissue levels of brain *myo*-inositol (*myo*-Ins) and taurine, and that TRAAK deletion reveals protective against ischemic injury. This beneficial effect designates this channel as a pharmacological target for the treatment against stroke.

## Materials and Methods

### Animals

Male C57Bl/6J mice (Charles River, L'Arbresle, France) were used as controls (Traak^+/+^ mice). Traak^−/−^ mice were a gift of the Institut de Pharmacologie Moléculaire et Cellulaire (IPMC, UMR 7275 CNRS, Valbonne, France). Traak^−/−^ mice were engineered as described previously [12]. Briefly, TRAAK genomic clones were isolated from a 129 mouse genomic BAC library by using a TRAAK cDNA probe. These clones were then subcloned into the pBluescript SK (Stratagene). After gene sequencing and mapping, targeting vectors and PCR primers were designed. KCNK4 gene disruption was obtained with stop codons inserted by an IRES-geo cassette, allowing interruption of TRAAK mRNA translation. After selection of the embryonic stem cell (ES) clone and linearization of the gene-targeting vector, chimeric males were generated by the injection of the targeted ES cells into C57Bl/6J blastocysts. These chimeric males were mated with female C57Bl/6J mice. Southern blot and PCR analysis of tail DNA from pups were used to assess the germline transmission. Heterozygous TRAAK-deficient mice were backcrossed with C57Bl/6J congenic mice over 11 generations. Traak^−/−^ mice were viable and healthy. They showed normal development and were fertile. The study was performed on Traak^−/−^ and Traak^+/+^ mice of the N10F2 backcross generation to C57Bl/6J congenic strain. All animals were 10–14 weeks old (25–28 g body weight) at the beginning of the *in vivo* MRI/MRS protocol.

### Ethics Statement

Animal studies were in agreement with the French guidelines for animal care from the French Ministry for Agriculture (Animal Rights Division), the European Council Directive 86/609/EEC of 24 November 1986, and approved by our institutional committee on Ethics in animal research. Surgery and imaging protocols were performed under gaseous anesthesia.

### Induction of focal cerebral ischemia

Focal ischemia was induced in mice anesthetized with 1–2% isoflurane in 50% O_2_: 50% N_2_O by tMCAO following a procedure previously described [25]. A midline incision was made at the neck, and the left common and external carotid arteries were isolated and ligated with a 4-0 silk suture (Ethicon, Brussels, Belgium). A Yasargil aneurysm clip (BMH31, Aesculap, Tuttlingen, Germany) was temporarily placed on the internal carotid artery. A 6-0 nylon monofilament (Ethicon), blunted at tip with an open flame, was introduced through a small incision into the common carotid artery and 13 mm distal to the carotid bifurcation for occlusion of the origin of the left MCA (supplementary Fig. S1). After one hour of ischemia, the thread was removed to allow reperfusion of the MCA territory [25]. During surgery, rectal temperature was maintained at 36±1°C with a homemade heating pad. Middle cerebral artery occlusion and reperfusion were assessed by magnetic resonance angiography.

### *In vivo* MR protocol

Gaseous anesthesia (2% isoflurane in 50% O_2_: 50% N_2_O) was used for imaging protocols. Mice were explored on a horizontal Bruker 47/30 AVANCE Biospec MR system operating at 4.7 T (Bruker, Karlsruhe, Germany) [26]. Traak^+/+^ and Traak^−/−^ mice were explored before tMCAO, during tMCAO, at immediate reperfusion (Im-RP), at 24 h post reperfusion (24 h-RP), and at 48 h post reperfusion (48 h-RP). After thread removal, each group of mice was divided into two subgroups, undergoing either the MRI or the MRS protocol to avoid prolonged anesthesia. The duration of each of these protocols was 45 minutes.

#### MRI protocol

Multi-slice axial transverse relaxation T_2_-weighted images and diffusion-weighted spin-echo echoplanar imaging used to map the apparent diffusion coefficient (ADC) were acquired with parameters already described [26]. Quantitative cerebral blood flow (CBF) maps were obtained from a single axial slice with a spin labeling technique [26]. Magnetic resonance angiography was performed on an 11.75 T vertical Bruker AVANCE 500WB wide-bore MR system [26], with a 3D-gradient echo time-of-flight sequence [26].

#### MRS protocol

^1^H-MRS brain spectra were obtained with the point resolved spatially localized spectroscopy (PRESS) sequence at two times of echo (TE) (16 and 135 ms). At a TE of 135 ms, the number of detectable metabolites is limited (choline-containing compounds, creatine+phosphocreatine, lactate) but overlap of metabolite signals is negligible and spectra show minimal baseline contribution because of the short transverse relaxation time (T_2_) of lipids and macromolecules. In addition, lactate signal may be unambiguously identified because of lipid signal suppression. However, signal loss resulting from metabolite transverse relaxation (T_2_) is considerable and may lead to an underestimation of metabolite levels. Moreover, *J*-coupled modulation may prevent the detection of many metabolites with multiplet resonance patterns (i.e. *myo*-inositol, taurine, glutamine and glutamate). On the contrary, a short TE (i.e.16 ms) affords the detection of a greater number of metabolites, including those bearing strongly coupled spin systems (i.e. *myo*-inositol, taurine, glutamine and glutamate) due to negligible *J*-coupled dephasing. Furthermore, the signal-to-noise ratio is high at short TE due to minimal T_2_ weighting, but baseline distortion caused by the underlying broad signals from macromolecules and lipids is not negligible [27], [28], [29].

Water signal suppression was achieved using a “variable power radiofrequency pulses with optimized relaxation delays” (VAPOR) sequence. A volume of interest (3.5^3^ mm^3^) was positioned in each brain hemisphere, comprising the caudate putamen and thalamus. Whole brain ^31^P-MRS was performed with a homemade surface coil (1 cm diameter) tuned to ^31^P (81 MHz) positioned over the skull, using a one-pulse sequence [26].

### MR data processing

Data were processed under IDL environment (Interactive Data Language Research System, Boulder, CO).

#### MRI Data

Brain volumetry was performed using T_2_-weighted images. The ADC maps were generated from the 3 sets of images recorded with increasing diffusion-weighting along orthogonal directions (ADCx, ADCy, ADCz). The average ADC was determined from the trace of the diffusion tensor [26]. CBF maps were calculated as described elsewhere [26]. Regional ADC and CBF values were evaluated as an average of pixel values in the cortex and in the caudate putamen+thalamus.

#### MRS Data

Data processing of ^1^H-MRS and ^31^P-MRS spectra was performed as described earlier [26]. The ^1^H-MRS spectra were referenced to creatine (3.04 ppm). The signal amplitudes stemming from total creatine (tCr = creatine+phosphocreatine), choline-containing compounds (Cho), glutamate+glutamine (Glx), lactate, *myo*-Ins, N-acetylaspartate (NAA) and taurine were calculated [26]. Results were expressed as ratios of the relative area of each metabolite signal to the sum of all metabolite signal areas (S) (S = NAA+tCr+Cho for spectra recorded at TE = 135 ms, S = NAA+tCr+Cho+Glx+*myo*-Ins+taurine for spectra recorded at TE = 16 ms).

^31^P-MRS chemical shifts were referenced to phosphocreatine (PCr) (−2.45 ppm). The signal amplitudes corresponding to PCr, inorganic phosphate (Pi), and α, β and γ-ATP were calculated. The chemical shift between Pi and PCr was used to calculate brain pH using the relationship: pH = pKa+log(δ-0.77/3.39-δ), where pKa is 6.8 and δ is the measured chemical shift of Pi in ppm relative to that of 85% phosphoric acid. Results were expressed as ratios of metabolites (PCr/Pi; β-ATP/Pi; PCr/β-ATP) [26].

### Statistical analysis

A non-parametric analysis was performed using the GraphPad 5.0a software (PRISM, La Jolla, CA). Within a same group, infarcted volumes, ADC values, CBF values and metabolic ratios were compared between the two hemispheres using the Wilcoxon test. The comparison of control values with those obtained at ischemia or reperfusion within each group (Traak^+/+^ or Traak^−/−^ mice) was performed using the Mann-Whitney test, as to the comparison between Traak^+/+^ and Traak^−/−^ mice at each time point. Values are reported as mean ± SEM. Significance was set to *p*<0.05.

## Results

### Incidence of TRAAK deletion on brain phenotype

**Figure 1:**
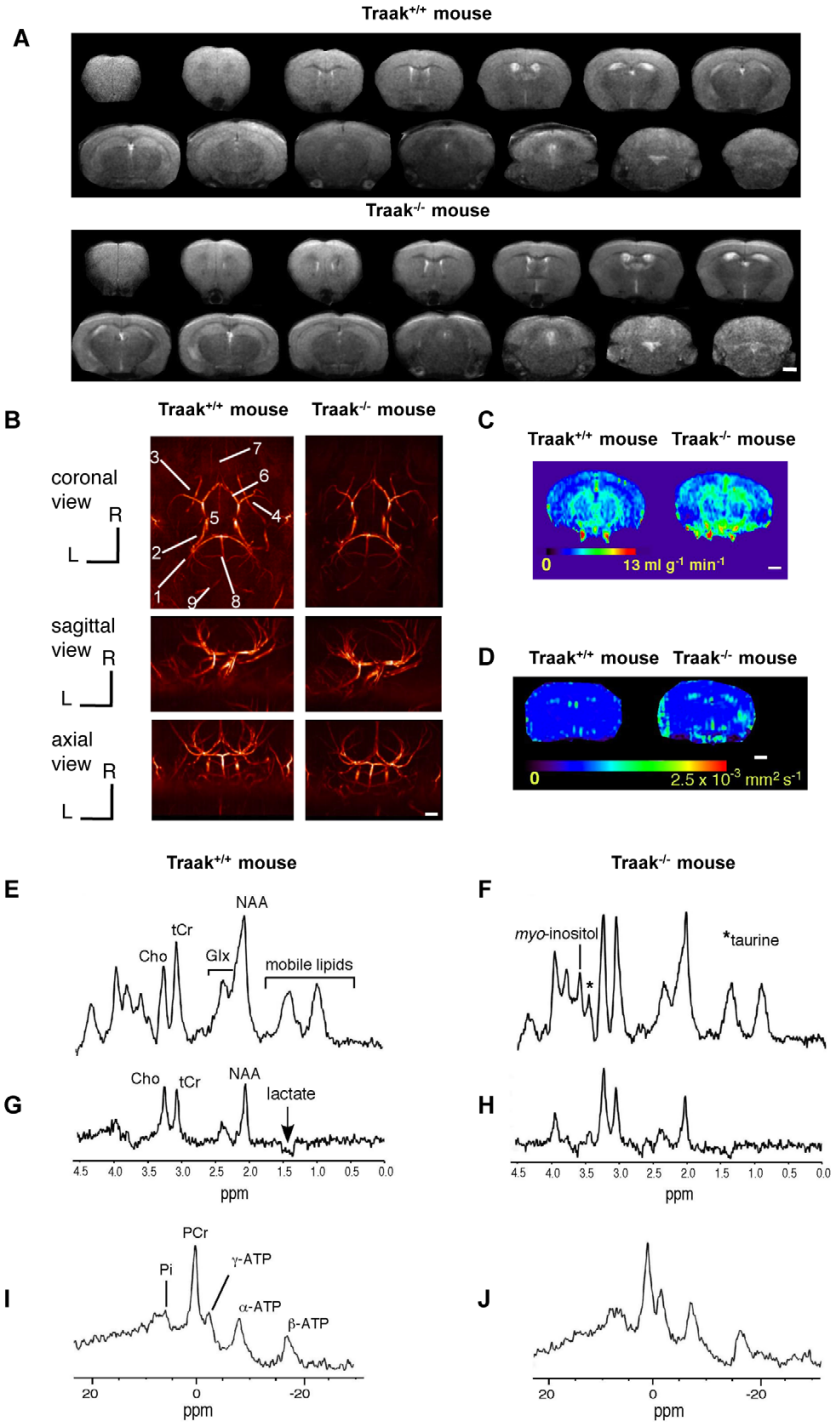
Characteristic brain MRI and MRS of Traak^+/+^ and Traak^−/−^ mice. (**A**) Typical axial T_2_-weighted images from a Traak^+/+^ and a Traak^−/−^ mouse. COR, cortex; H, hippocampus; S, striatum; V, ventricles. (**B**) Axial, coronal and sagittal maximum intensity projections of a 3D time-of-flight angiogram of a Traak^+/+^ and a Traak^−/−^ mouse. C: caudal; D: dorsal; R rostral; L: left orientations. 1: common carotid artery; 2: internal carotid artery; 3: external carotid artery; 4: middle cerebral artery; 5: Willis circle; 6: anterior cerebral artery; 7: ophthalmic artery; 8: basilar artery; 9: vertebral artery. (**C**) Representative perfusion maps from a Traak^+/+^ and Traak^−/−^ mouse. (**D**) Representative ADC maps from a Traak^+/+^ and a Traak^−/−^ mouse. (**E**) and (**F**) Representative cerebral ^1^H-MRS (TE = 16 ms) from a Traak^+/+^ and Traak^−/−^ mouse. (**G**) and (**H**) Representative cerebral ^1^H-MRS (TE = 135 ms) from a Traak^+/+^ and Traak^−/−^ mouse. (**I**) and (**J**) Typical brain ^31^P-MRS spectra from a Traak^+/+^ and Traak^−/−^ mouse (PME: phosphomonoesters). Scale bars = 1 mm.

Brain morphology (Fig. 1A) was equivalent in Traak^+/+^ and Traak^−/−^ mice . There were no significant volumetric differences between groups (data not shown). The anatomy of arteries was identical without any flow disturbance or difference in vessel architecture (Fig. 1B). CBF values (Fig. 1C) were similar in cortex (Traak^+/+^ mice: 2.67±0.09 ml g^−1^ min^−1^, n = 13, Traak^−/−^ mice: 2.75±0.22 ml g^−1^ min^−1^, n = 15) and caudate putamen+thalamus (Traak^+/+^ mice: 4.02±0.23 ml g^−1^ min^−1^, n = 13, Traak^−/−^ mice: 4.09±0.29 ml g^−1^ min^−1^, n = 15). ADC values (Fig. 1D) were similar in cortex (Traak^+/+^ mice: 8.03±0.26×10^−4^ mm^2^ s^−1^, n = 18, Traak^−/−^ mice: 8.31± 0.24×10^−4^ mm^2^ s^−1^, n = 16) and caudate putamen+thalamus (Traak^+/+^ mice: 8.78±0.71×10^−4^ mm^2^ s^−1^, n = 18, Traak^−/−^ mice: 8.36±0.31×10^−4^ mm^2^ s^−1^, n = 16).

^1^H-MRS was performed in the left and right hemispheres of each animal and showed no difference between the two hemispheres. Consequently, the mean value of each metabolite ratio was used for comparison between Traak^+/+^ and Traak^−/−^ mice (Table 1). At short TE, significantly higher levels of taurine/S and *myo*-inositol/S were measured in Traak^−/−^ mice compared to Traak^+/+^ mice (+327% for taurine/S and +246% for *myo*-inositol/S) (Figs. 1E–F and Table 1). At long TE, no difference was observed (Figs. 1G–H and Table 1). ^31^P-MRS showed similar pH and energy metabolite levels in Traak^+/+^ and Traak^−/−^ mice (Figs. 1I–J and Table 1).

**Table 1:** Comparative brain metabolite analysis of Traak^+/+^ and Traak^−/−^ mice.

Metabolite ratio	Traak^+/+^ mice	Traak^−/−^ mice	Statistics
**^1^H-MRS (TE = 16 ms)**	(*n* = 11)	(*n* = 15)	
**NAA/S**	0.31±0.02	0.27±0.01	
**tCr/S**	0.21±0.01	0.19±0.01	
**Cho/S**	0.13±0.01	0.14±0.01	
**Glx/S**	0.32±0.03	0.31±0.02	
**taurine/S**	0.010±0.003	0.036±0.003	*p*<0.0001
***myo*** **-inositol/S**	0.026±0.004	0.064±0.005	*p*<0.0001
**(lipids+lactate)/S**	0.38±0.03	0.36±0.02	
**^1^H-MRS (TE = 135 ms)**	(*n* = 12)	(*n* = 15)	
**NAA/S**	0.33±0.01	0.32±0.01	
**tCr/S**	0.33±0.00	0.32±0.01	
**Cho/S**	0.33±0.01	0.36±0.01	
**lactate/S**	0.08±0.01	0.06±0.01	
**^31^P-MRS**	(*n* = 10)	(*n* = 10)	
**PCr/β-ATP**	2.06±0.19	2.10±0.28	
**PCr/Pi**	5.48±0.38	6.29±0.02	
**β-ATP/Pi**	2.93±0.41	3.36±0.43	
**pH**	7.12±0.01	7.10±0.02	

### The infarction volume is smaller in Traak^−/−^ mice after tMCAO

**Figure 2:**
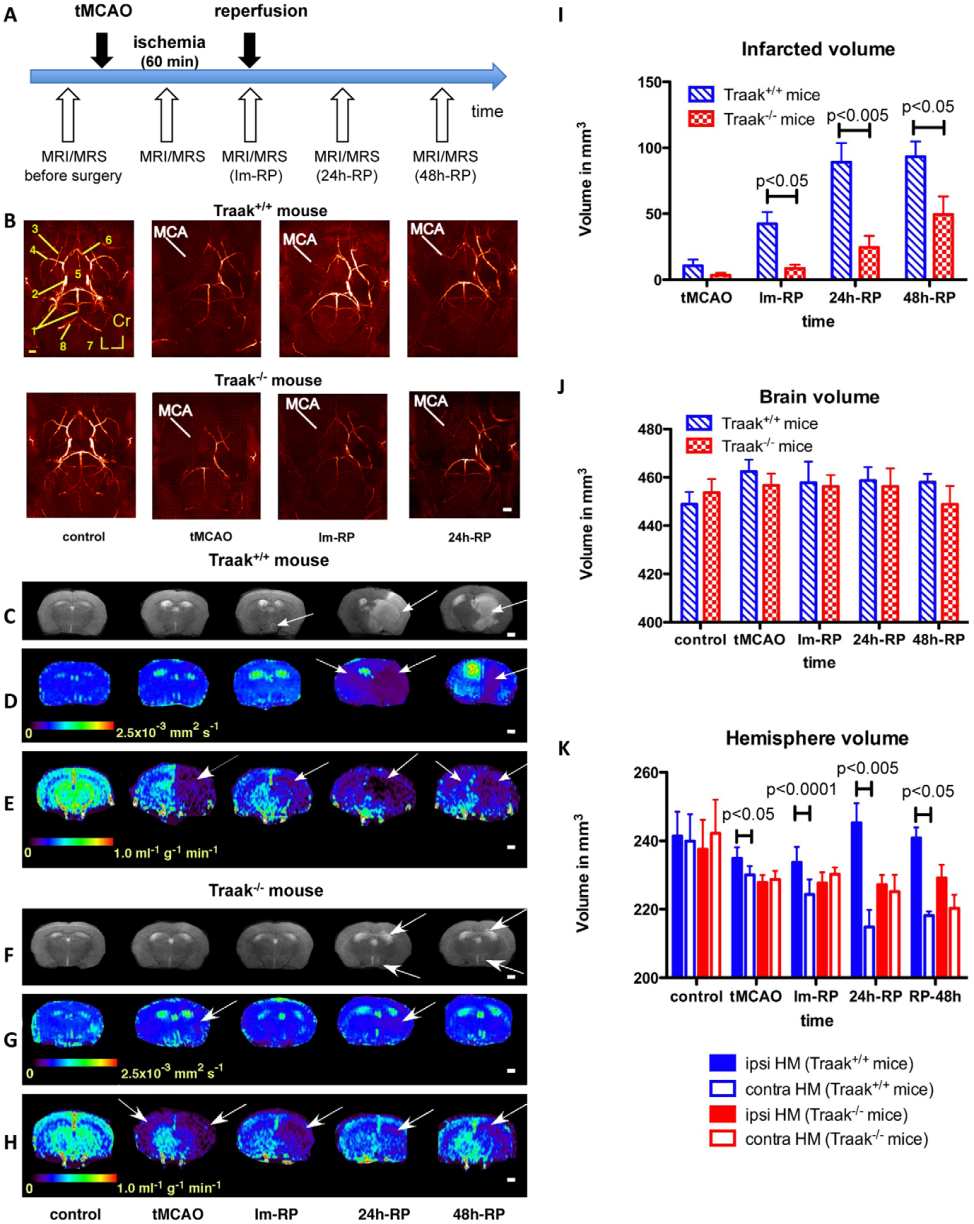
Structural and functional changes during tMCAO and following reperfusion in Traak^+/+^ and Traak^−/−^ mice. (**A**) *In vivo* MRI/MRS protocol. (**B**) Axial, coronal and sagittal maximum intensity projections of a 3D time-of-flight angiogram of a Traak^+/+^ and a Traak^−/−^ mouse before ischemia, during tMCAO and after reperfusion. The angiograms show an absence of signal in the MCA during ischemia and a flow recovery after reperfusion in both Traak^+/+^ and Traak^−/−^ mice. (**C**), (**D**) and **E**) T_2_-weighted MRI, ADC maps, and perfusion maps of a Traak^+/+^ mouse before ischemia, during tMCAO and at reperfusion. Although T_2_-weighted MRI and ADC map show an extensive lesion in the ipsilateral hemisphere (arrows) from 24 h-RP on, the lesion was already visible at tMCAO, but at an anatomic level not shown here. Perfusion maps show a strong reduction in CBF during tMCAO and following reperfusion in both hemispheres. (**F**), (**G**) and (**H**) T_2_-weighted MRI, ADC and perfusion maps of a Traak^−/−^ mouse before ischemia, during tMCAO and at reperfusion. Note the small lesion on T_2_-weighted MRI and ADC maps (arrows) and the total mismatch between ADC and CBF maps. (**I**) Temporal course of the infarcted volume, (**J**) of the brain volume, (**K**) and of the hemisphere volume in both Traak^+/+^ and Traak^−/−^ mice. Control: *n* = 26 and 17, tMCAO: *n* = 10 and 14, Im-RP: *n* = 9 and 10, 24 h-RP: *n* = 9 and 9, 48 h-RP: *n* = 3 and 6 for Traak^+/+^ and Traak^−/−^ mice respectively. Abbreviations: contra, contralateral; HM, hemisphere; ipsi, ipsilateral.

The animals were explored before, during and after tMCAO (Fig. 2A). The surgical procedure was validated by magnetic resonance angiography (Fig. 2B). In Traak^+/+^ mice, the ischemic lesion was visible at Im-RP (Fig. 2C) and extended to the ipsilateral hemisphere at 24 h-RP, causing an important midline shift (Figs. 2C–D). Meanwhile, CBF was reduced in both hemispheres despite MCA reperfusion (Fig. 2E). In Traak^−/−^ mice, the ischemic lesion was hardly detectable before 24 h-RP (Figs. 2F–G), although CBF remained low after reperfusion in the ipsilateral hemisphere (Fig. 2H). The infarction volume (Fig. 2I) was significantly lower in Traak^−/−^ mice at Im-RP (Traak^+/+^ mice: 42.40±8.88 mm^3^, Traak^−/−^ mice: 8.57±2.82 mm^3^, p<0.05), at 24 h-RP (Traak^+/+^ mice: 89.01±14.68 mm^3^, Traak^−/−^ mice: 24.40±8.94 mm^3^, p<0.005), and at 48 h-RP (Traak^+/+^ mice: 93.17±11.67 mm^3^, Traak^−/−^ mice: 49.47±13.54 mm^3^, p<0.05). Brain volumes were not different in Traak^+/+^ and Traak^−/−^ mice (Fig. 2J). However, an expansion of the ipsilateral hemisphere occurred at the expense of the contralateral hemisphere in Traak^+/+^ mice (Figs. 2C, 2K) indicating a crushing of this structure. This difference was always significant (tMCAO: p<0.05; Im-RP: p<0.0001; 24 h-RP: p<0.005; 48 h-RP: p<0.05). Conversely, Traak^−/−^ mice did not show any variation in hemisphere volume during the follow-up (Fig. 2K).

### Cellular edema is less developed in Traak^−/−^ mice

**Figure 3:**
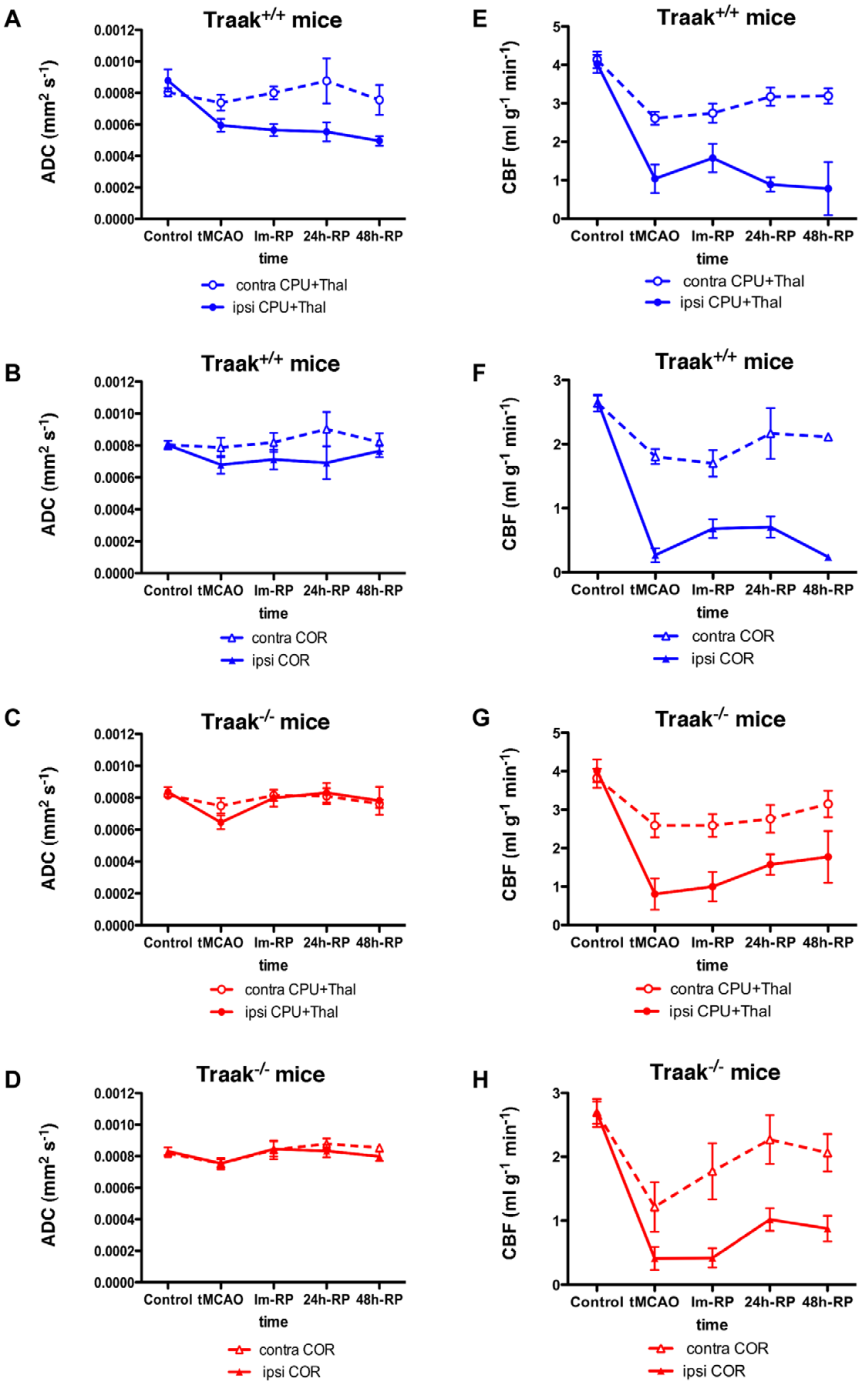
Temporal course of ADC and CBF in Traak^+/+^ and Traak^−/−^ mice. (**A**), (**C**) ADC values in the ipsilateral hemisphere of Traak^+/+^ and Traak^−/−^ mice respectively. (**B**), (**D**) CBF values in the ipsilateral hemisphere of Traak^+/+^ and Traak^−/−^ mice respectively. (**E**), (**G**) ADC values in the contralateral hemisphere of Traak^+/+^ and Traak^−/−^ mice respectively. (**F**), (**H**) CBF values in the contralateral hemisphere of Traak^+/+^ and Traak^−/−^ mice respectively. ADC maps, control: *n* = 18 and 16, tMCAO: *n* = 13 and 7, Im-RP: *n* = 10 and 8, 24 h-RP: *n* = 8 and 12, 48 h-RP: *n* = 4 and 6 for Traak^+/+^ and Traak^−/−^ mice respectively. CBF maps, control: *n* = 13 and 15, tMCAO: *n* = 6 and 4, Im-RP: *n* = 6 and 4, 24 h-RP: *n* = 4 and 7, 48 h-RP: *n* = 3 and 4 for Traak^+/+^ and Traak^−/−^ mice respectively. Abbreviations: contra, contralateral; COR, cortex; CPU, caudate putamen; ipsi, ipsilateral; Thal, thalamus.

Traak^+/+^ mice presented a marked drop of ADC values upon occlusion in the ipsilateral caudate putamen+thalamus (tMCAO, Im-RP, and 24 h-RP: *p*<0.001; 48 h-RP: *p*<0.05), when compared to the control values (Figs. 2D and 3A). The ADC value was lower in the ipsilateral cortex only during tMCAO (*p*<0.05). The contralateral structures were not significantly affected during the follow-up (Fig. 3B). Comparison of the two hemispheres at the same time points showed that ADC values were lower in the ipsilateral cortex except for 48 h-RP (tMCAO, Im-RP, and 24 h-RP: *p*<0.05) (Figs. 3A–B). From Im-RP, ADC values became lower in the ipsilateral caudate putamen+thalamus when compared to the contralateral structure at the same time point (Im-RP: *p*<0.0001; 24 h-RP and 48 h-RP: *p*<0.05).

Traak^−/−^ mice had normal ADC values (Figs. 2G and 3C–D), apart from a transient decrease in the ipsilateral caudate putamen+thalamus at tMCAO (*p*<0.05). However, the comparison of the ipsilateral and contralateral hemispheres revealed that ADC values were lower in the ipsilateral caudate putamen+thalamus at tMCAO (*p*<0.05) and in the ipsilateral cortex at 24 h-RP only (*p*<0.01).

### Brain microcirculation is less damaged in Traak^−/−^ mice and recovers more rapidly after tMCAO

Traak^+/+^ mice showed a dramatic CBF reduction upon occlusion in both hemispheres, persisting after reperfusion (Figs. 2E and 3E–F) and remaining lower than normal in the ipsilateral cortex (tMCAO: *p*<0.0005; Im-RP: *p*<0.001; 24 h-RP: *p*<0.005; 48 h-RP: *p*<0.001), and the ipsilateral caudate putamen+thalamus (tMCAO: *p*<0.0005; Im-RP: *p*<0.01; 24 h-RP: *p*<0.005; 48 h-RP: *p*<0.05). Perfusion of the contralateral hemisphere was altered during tMCAO and at Im-RP (contralateral cortex: Im-RP: *p*<0.05; contralateral caudate putamen+thalamus during tMCAO and at Im-RP: *p*<0.05) but recovered thereafter. CBF values tended to be lower in the ipsilateral cortex until 24 h-RP (tMCAO: *p*<0.01, Im-RP: *p* = 0.0625). At 48 h-RP, the CBF in the contralateral caudate putamen+thalamus and the contralateral cortex represented 77% and 80% of the control value respectively, whereas in the ipsilateral caudate putamen+thalamus and the ipsilateral cortex it was only 19% and 9% of the normal value respectively.

Traak^−/−^ mice presented a drop of CBF during and after tMCAO in both hemispheres (Figs. 2H and 3G–H). The CBF value was lower in the ipsilateral cortex during tMCAO, Im-RP and at 24 h-RP (*p*<0.005), and in the ipsilateral caudate putamen+thalamus during tMCAO and Im-RP (*p*<0.005), and at 24 h-RP (*p*<0.0005). In contrast to Traak^+/+^ mice, ipsilateral structures recovered at 48 h-RP in Traak^−/−^ mice. Contralateral structures showed reduced perfusion during tMCAO and Im-RP only (contralateral cortex: tMCAO, *p*<0.05; contralateral caudate putamen+thalamus during tMCAO and at Im-RP: *p*<0.05). At 48 h-RP, the CBF in the contralateral caudate putamen+thalamus and in the contralateral cortex represented 82% and 77% of the control value respectively, whereas in the ipsilateral caudate putamen+thalamus and the ipsilateral cortex it was 44% and 33% of the normal value respectively. These results reveal a better recovery of the microcirculation in Traak^−/−^ mice.

### Brain metabolic ischemic profiles are different in Traak^+/+^ and Traak^−/−^ mice

**Figure 4:**
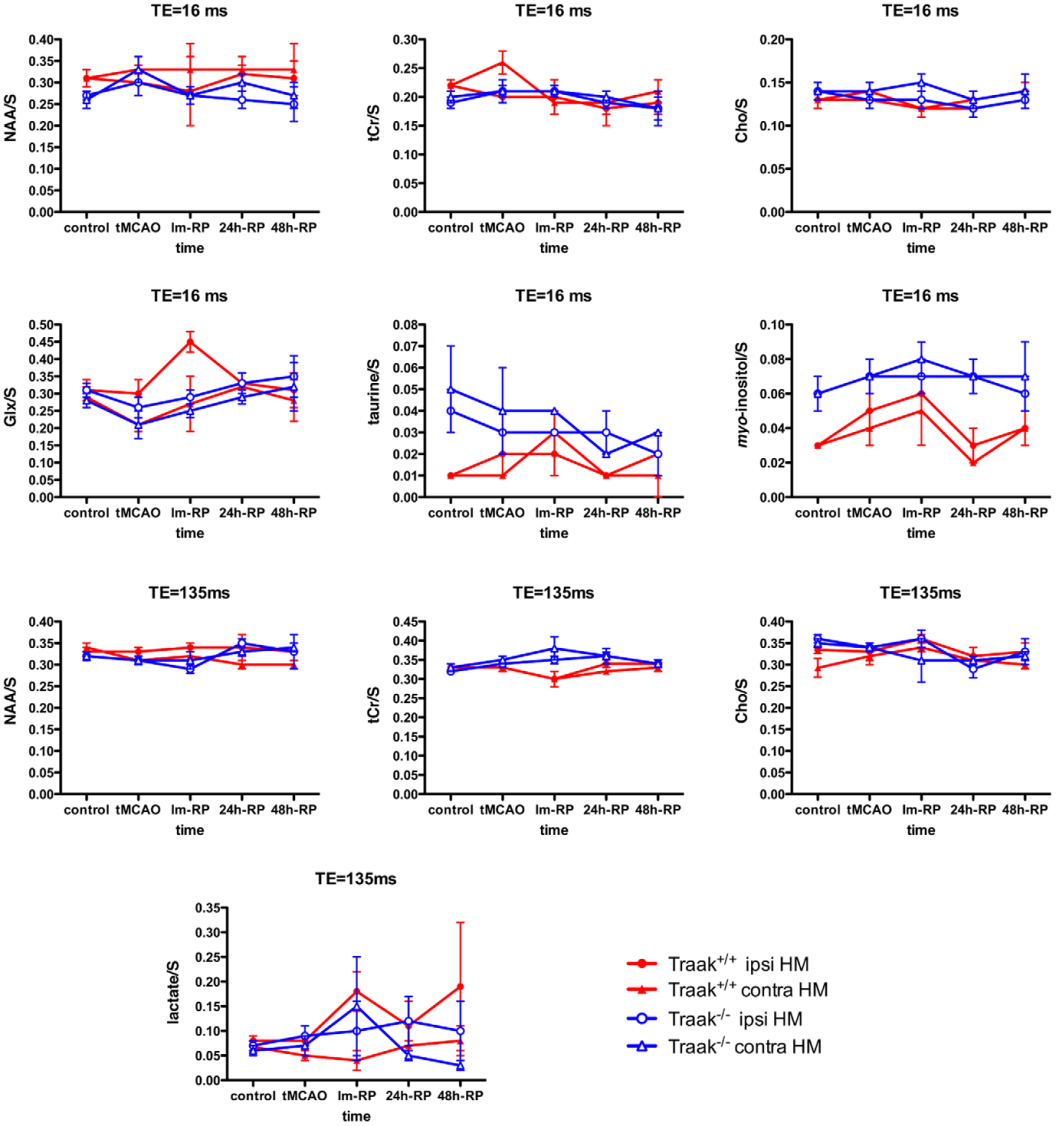
Time evolution of major brain metabolites in Traak^+/+^ and Traak^−/−^ mice. Brain metabolic ratios as determined by ^1^H-MRS in ipsilateral and contralateral caudate putamen+thalamus. TE = 16 ms: control: *n* = 11 and 15, tMCAO: *n* = 6 and 7, Im-RP: *n* = 4 and 6, 24 h-RP: *n* = 6 and 9, 48 h-RP: *n* = 4 and 3 for Traak^+/+^ and Traak^−/−^ mice respectively. ^1^H-MRS TE = 135 ms: control: *n* = 12 and 15, tMCAO: *n* = 7 and 6, Im-RP: *n* = 4 and 6, 24 h-RP: *n* = 5 and 10, 48 h-RP: *n* = 4 and 3 for Traak^+/+^ and Traak^−/−^ mice respectively. Abbreviations: contra, contralateral; HM, hemisphere; ipsi, ipsilateral.

At short TE, Traak^+/+^ mice showed a strong increase in *myo*-inositol/S in the ipsilateral hemisphere during tMCAO and at Im-RP (*p*<0.05), and a swift increase in Glx/S at Im-RP (*p* = 0.05) associated with a concomitant rise in taurine/S albeit non-significant. Traak^−/−^ showed metabolic variations only in the contralateral hemisphere consisting in an increase in NAA/S at tMCAO (*p*<0.05) and a marked reduction in taurine/S at 24 h-RP (*p*<0.05) (Fig. 4).

At long TE, Traak^+/+^ mice showed a decrease in NAA/S in the contralateral hemisphere at 48 h-RP (*p*<0.05), a drop in tCr/S in the ipsilateral hemisphere at Im-RP (*p*<0.05) (Fig. 4) and a burst of lactate at Im-RP statistically significant in the ipsilateral hemisphere (*p*<0.05). Conversely, Traak^−/−^ mice showed a progressive increase in tCr/S in the ipsilateral hemisphere (tMCAO, Im-RP, and 24 h-RP: *p*<0.05) accompanied by a decrease in Cho/S at 24 h-RP (*p*<0.05). A non-significant increase in lactate was observed in the ipsilateral hemisphere only at Im-RP (Fig. 4).

### Brain pH and energetic metabolism are preserved in Traak^−/−^ mice

**Figure 5:**
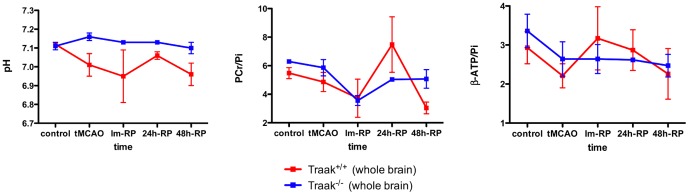
Time evolution of brain energy metabolites and pH determined by ^31^P-MRS of the whole brain. Control: *n* = 10 and 10, tMCAO: *n* = 10 and 7, Im-RP: *n* = 4 and 4, 24 h-RP: *n* = 5 and 3, 48 h-RP: *n* = 3 and 3 for Traak^+/+^ and Traak^−/−^ mice respectively.

During tMCAO and after reperfusion, Traak^+/+^ mice showed a strong decrease in brain pH (tMCAO: *p*<0.005; Im-RP: *p*<0.05). A reduction in PCr/Pi was observed at 48-RP in Traak^+/+^ mice (*p*<0.05), and of ATP/Pi, although non-significant. Traak^−/−^ mice showed a non-significant decrease in PCr/Pi and ATP/Pi, and no pH fluctuation during the follow-up (Fig. 5).

## Discussion

Although the cellular localization, the regulatory mechanisms and the neuroprotective effects of TREK channels indicate their involvement in neuroglial coupling [4], [6], TRAAK functions in brain are still unclear. Here, we provide the first *in vivo* evidence establishing a link between TRAAK and neurometabolism. Furthermore, we report that TRAAK deletion is protective against cerebral transient focal ischemia.

### Deletion of TRAAK alters brain levels of taurine and *myo*-inositol

The striking feature of Traak^−/−^ mice compared to Traak^+/+^ mice is their higher level of taurine and *myo*-inositol, two important mediators of neural cell volume regulation [30]. Taurine is an aminoacid involved in membrane stabilization, neurotransmission, and neuroprotection [31], [32], and modulates the activity of a wide range of ion channels in brain including some potassium channels [32], [33]. Brain taurine mainly derives from blood flow and hepatic synthesis, but can be produced by astrocytes [34] and to a lesser extent by neurons [35]. Taurine specific transporters (TauT) are heterogeneously distributed across brain regions and among neural cell populations [36]. *Myo*-inositol, a polyol present at low levels in neurons [37] but highly concentrated in astrocytes [38] is a major brain osmolyte [39]. Our results highlight an unexpected control of neuro-glial metabolism by TRAAK affecting two quantitatively important organic osmolytes. Under physiological and pathological conditions, cell shrinkage or swelling may occur. These processes can be counteracted by regulatory volume increase or decrease through the gain or loss of osmotically active compounds such as electrolytes (Na^+^, K^+^, Cl^−^) or organic molecules called “non-perturbing” solutes (*myo*-inositol, taurine or betaine) [40], [41], [42], [43]. Whereas large shifts in electrolytes may affect membrane potential, structural integrity, and compromise cellular survival, cells are capable to withstand important variations in non-perturbing osmolytes without any damage [40], [41], [42], [43]. Our results demonstrate that TRAAK, an effector in cell volume regulation [2], [43], contributes to the control of non-perturbing osmolytes. The higher levels of taurine and *myo*-inositol in Traak^−/−^ mice presumably relate to changes in intracellular K^+^ affecting the transport, efflux or synthesis rate of these osmolytes. Indeed, the expression of genes encoding taurine (TauT) and *myo*-inositol (SMIT) transporters is coupled to osmolarity [44] and ionic strength in neurons and astrocytes [45], [46], [47]. An increase in intracellular K^+^ resulting from TRAAK deletion might lead to compensatory mechanisms involving other K_2_P channels such as TREK-1 and TREK-2 in neurons. In astrocytes, which are involved in K^+^ uptake and buffering [3], these compensatory mechanisms could possibly involve TREK-2, TASK-1, and TASK-3 that have been identified in these cells [3]. However, because K_2_P channels show only weak inward rectification, it is likely that other channels such as inward rectifier potassium channels (Kir) or the Na^+^/K^+^/Cl^−^ co-transporter could contribute to these mechanisms. Another possibility would consist in the redistribution of other ions such as Na^+^, and Cl^−^ in the intracellular and extracellular compartments in response to the shift in intracellular K^+^. To our knowledge, neither the changes in electrolyte levels, nor the possible compensation mechanisms that may affect other K_2_P in neural cells from Traak^−/−^ mice have been examined so far [48]. Our results suggest that TRAAK deletion may elicit a shift in intracellular K^+^ influencing the concentration of taurine in neurons *via* both import and synthesis since neurons are capable to activate taurine synthesis under hypertonic conditions [35]. However, we cannot exclude the possibility that TRAAK deletion may also impact taurine level in astrocytes, since neurons import not only hypotaurine, a precursor of taurine, but also taurine from astrocytes [38], [49]. The increase in *myo*-inositol could similarly be linked to the shift in intracellular K^+^ resulting from TRAAK deletion. Although *myo*-inositol is predominantly found in astrocytes, mRNA of the SMIT co-transporter for *myo*-inositol has been found in almost all neurons [50]. This finding suggests that the level of *myo*-inositol in neurons may vary in response to changes in tonicity. As for taurine, we cannot rule out a possible increase in glial *myo*-inositol as a consequence of astrocyte involvement in ionic buffering.

### Traak^−/−^ phenotype is protective against cerebral ischemia

Altogether, the smaller infarct, the modest cellular edema, the less damaged microcirculation, and the preserved brain energy indicate that Traak^−/−^ phenotype is protective against ischemia.

Traak^+/+^ mice displayed a significant disruption of CBF in the ipsilateral hemisphere that persisted for 2 days after ischemia, whereas the contralateral hemisphere recovered after recirculation. Hypoperfusion in the contralateral hemisphere has already been reported in humans and in animal models of focal ischemia [51], [52]. This phenomenon termed diaschisis would reflect depressed metabolic and synaptic activity in remote brain regions. In addition, Traak^+/+^ mice showed an important decline of ADC reflecting acute ultrastructural changes such as cytotoxic edema affecting mostly astrocytes, extracellular compartment restriction, and neuronal shrinkage [53]. Cytotoxic edema results from anoxic depolarization after the failure of Na^+^/K^+^-ATPases to maintain membrane potential upon ATP depletion, which leads to an accumulation of intracellular electrolytes [54]. During ischemia, Traak^+/+^ mice showed important depletion of PCr/Pi accompanied by lactate production and marked acidification. Despite a partial recovery after reperfusion, pH and PCr/Pi markedly declined at 48 h-RP, probably because of delayed cell death after reoxygenation due to mitochondrial impairment [55]. Energy failure and acidification were presumably underestimated, as it was not possible to isolate the contribution pertaining only to the lesion when performing ^31^P-MRS. The pH fall results from anaerobic lactate production and H^+^ accumulation [56]. Tissue acidification is a well-known trigger of neuronal cell death under ischemia [57]. The decrease of the neuronal marker NAA in Traak^+/+^ mice, indicates neuronal damage or loss due to mitochondrial impairment, energy failure and excitoxicity [56]. Indeed, NAA synthesis is mitochondrial and coupled to glucose metabolism [58]. Hence, NAA decrease together with the reduction in tCr/S and PCr/Pi points to a mitochondrial dysfunction due to oxygen-glucose deprivation [58]. The severity of the NAA decrease has a prognostic value in experimental and human cerebral stroke [23], [59].

In Traak^−/−^ mice, diffusion restriction was almost undetectable during ischemia, and returned to normal after reperfusion. As for Traak^+/+^ mice, contralateral hypoperfusion resolving with recirculation was observed. There was a total mismatch between MR angiography, diffusion-weighted MRI and perfusion-weighted MRI in these mice. Angiography confirmed the occlusion of MCAO after surgery, and blood recirculation after thread withdrawal. CBF was significantly reduced during ischemia and tended to recover after reperfusion, whereas ischemic lesions were almost undetectable on ADC maps. Interestingly, a similar finding has been observed in patients diagnosed with minor ischemic strokes [60], [61], [62]. This result suggests that the CBF, although significantly reduced, was still above the critical threshold leading to non-reversible energy failure. This hypothesis seems corroborated by the observation of preserved brain energy metabolism and pH in these mice. Moreover, Traak^−/−^ mice did not display the classical pattern of early metabolic alterations elicited by ischemia and consisting in an increase in lactate and a decrease of NAA, creatine and phosphocreatine [23], [59], [63], [64]. Actually, a reversible increase in NAA/S and tCr/S occurred during tMCAO, probably reflecting reduced degradation and/or release of these compounds, since accrued synthesis of NAA and creatine seems unlikely in a context of glucose and oxygen deprivation.

### Traak^+/+^ and Traak^−/−^ mice exhibit different osmolyte profiles in response to cerebral ischemia induced by tMCAO

In Traak^−/−^ mice, taurine decreased progressively from ischemia, as previously reported in mice undergoing short (10 minutes) tMCAO [59], a finding consistent with an ischemia-evoked efflux of taurine, a protective mechanism counteracting excitotoxicity and allowing regulatory volume decrease to attenuate cellular edema [65]. The released taurine was then washed-out by the microcirculation after reperfusion. Conversely, taurine was not decreased in Traak^+/+^ mice, probably because microcirculation was too severely impaired to eliminate the extra-cellular aminoacid. The apparent response of Traak^+/+^ mice to ischemia consisted in a significant increase in *myo*-inositol/S at tMCAO and reperfusion associated with an increase in Glx/S reflecting a rise in glutamine due to glutamate detoxification [59]. Elevated *myo*-inositol has been reported in patients with cerebral ischemia and attributed to the replacement of electrolytes by *myo*-inositol in swollen astrocytes [66]. Another possibility could be linked to neuronal osmoregulation since SMIT expression is increased in neurons after focal cerebral ischemia [67].

Traak^−/−^ mice better resistance to cellular edema appears related to the physiologically higher levels of *myo*-inositol and especially of taurine which also has cytoprotective properties [31]. Taurine protects neural cells from excitotoxicity by preventing the expression of caspases triggered by ischemia [68], [69], [70], [71], [72] and by reducing extracellular glutamate release evoked by ischemia [73], [74] whereas taurine deficient astrocytes show less efficient cell volume regulation upon osmotic challenge [75]. In experimental cerebral ischemia, taurine administration protects microcirculation, enhances ATP level, down-regulates Bax, up-regulates Bcl-xL, and diminishes caspase-3 mediated apoptosis. This modulation of mitochondrial activity and cell death concurs to reduce the infarct volume [70], [72], [76], [77]. Our results on Traak^−/−^ mice are consistent with these previous studies demonstrating the neuroprotective effects of taurine under ischemia.

The high levels of taurine and *myo*-inositol, the tight regulation of astrocytic, neuronal and microvascular functions during stroke, and the better recovery after reperfusion describe an enhanced protective response pathway of the neurovascular unit to ischemia. The effects of the deletion of neuronal [7] and vascular [10] TRAAK channels suggest that TRAAK contributes to the regulation of organic osmolytes. The observation of a more important infarct in Traak^+/+^ mice could be linked to the morphometric changes and cytoskeleton reorganization accompanying cell swelling under ischemia [78], [79]. These modifications may induce membrane stretching, a process known to activate TRAAK channels and to increase their sensitivity to arachidonic acid [14]. TRAAK activation by high concentrations of arachidonic, as those produced under ischemia, could be highly detrimental because the opening of the channels would progressively drain K^+^ out of the neurons [16].

### Pharmacological modulation of TRAAK for brain stroke treatment

Our results are consistent with the hypothesis that TRAAK inhibition could be neuroprotective under certain conditions [80]. Activation of TRAAK channels would be the expected mechanism to provide neuroprotection since α-linolenic acid and riluzole, two non-selective activators of TREK family channels, were found protective against focal cerebral ischemia [25]. The beneficial effects observed in C56BL/6J mice induced for tMCAO and treated with these agents were attributed to the activation of TREK-1 and TRAAK channels, although they were probably involving other K^+^ channels, voltage-dependent Na^+^ and Ca^2+^ channels [13]. Indeed, riluzole not only inhibits glutamatergic pathways, but also blocks voltage-gated K^+^ channels [81], calcium channels [82], [83], persistent Na^+^ currents (*I*_Na,p_) [84] and modulates brain sodium channel Nav1.1 under acute cerebral ischemia [85]. In addition, α-linolenic acid can bind to voltage sensitive Na^+^ channels [86]. The precise contribution of TRAAK channels activation to the protective effects observed under focal ischemia was undetermined. On the other hand, sipatrigine, a neuroprotective agent and potent inhibitor of TREK-1 and TRAAK channels [80] was found neuroprotective under focal and global ischemia [87]. In addition to its action on TREK-1 and TRAAK channels, sipatrigine blocks sodium channels including *I*_Na,p_, and voltage-gated Ca^2+^ channels, and inhibits glutamate release [88]. Although an important limitation of these pharmacological approaches is the use of non-selective activators or inhibitors, these studies lead to the apparently contradictory conclusion that neuroprotection mediated by TREK channels could be achieved *via* both activation and inhibition. Our findings on mice lacking TRAAK indicate that specific blockade of this channel could represent a strategy to confer neuroprotection under ischemia. Since increased brain taurine appears as one the main positive consequences of TRAAK deletion, the administration of this aminoacid should theoretically reproduce, at least in part, the neuroprotective effects observed upon ischemia in mice lacking TRAAK. Because administration of taurine failed to significantly increase the level of this aminoacid in the human brain [31], we suggest that the cerebroprotective effects resulting from TRAAK deletion should prompt the search for selective blockers of TRAAK [89] for preventive or therapeutic treatments against stroke

## Supporting Information

Figure S1 **Anatomical scheme of the transient Middle Cerebral Artery Occlusion Model with the intraluminal filament technique in mouse.**
(TIF)
